# A Dynamic 3D Aggregate-Based System for the Successful Expansion and Neural Induction of Human Pluripotent Stem Cells

**DOI:** 10.3389/fncel.2022.838217

**Published:** 2022-03-03

**Authors:** Cláudia C. Miranda, Michael L. Akenhead, Teresa P. Silva, Michael A. Derr, Mohan C. Vemuri, Joaquim M. S. Cabral, Tiago G. Fernandes

**Affiliations:** ^1^Department of Bioengineering and iBB – Institute for Bioengineering and Biosciences, Instituto Superior Técnico, Universidade de Lisboa, Lisbon, Portugal; ^2^Associate Laboratory i4HB-Institute for Health and Bioeconomy at Instituto Superior Técnico, Universidade de Lisboa, Lisbon, Portugal; ^3^Thermo Fisher Scientific, Cell Biology, Life Sciences Solutions, Frederick, MD, United States; ^4^Instituto de Medicina Molecular João Lobo Antunes, Faculdade de Medicina, Universidade de Lisboa, Lisbon, Portugal

**Keywords:** human induced pluripotent stem cells, expansion, scale-up, neural induction, dopaminergic differentiation

## Abstract

The demand for large cell numbers for cellular therapies and drug screening applications requires the development of scalable platforms capable of generating high-quality populations of tissue-specific cells derived from human pluripotent stem cells (hPSCs). Here, we studied the ability of Gibco StemScale PSC Suspension Medium to promote the efficient expansion of hPSC cultures as aggregates grown in suspension. We tested human induced pluripotent stem cell (hiPSC) growth in 6-well plates (on orbital shaker platforms) and single-use vertical-wheel bioreactors for a total of three consecutive passages. Up to a 9-fold increase in cell number was observed over 5 days per passage, with a cumulative fold change up to 600 in 15 days. Additionally, we compared neural induction of hiPSCs by using a dual SMAD inhibition protocol with a commercially available neural induction medium, which can potentially yield more than a 30-fold change, including neural progenitor induction and expansion. This system can also be adapted toward the generation of floor plate progenitors, which yields up to an 80-fold change in cell number and generates FOXA2-positive populations. In summary, we developed platforms for hiPSC expansion and neural induction into different brain regions that provide scalability toward producing clinically relevant cell numbers.

## Introduction

Human pluripotent stem cells (hPSCs) have long been in the spotlight due to their potential for self-renewal and ability to virtually differentiate into any cell from the three embryonic germ layers: mesoderm, ectoderm, and endoderm (Thomson et al., 1998). Moreover, since the derivation of human induced pluripotent stem cells (hiPSCs), the possibility of circumventing ethical concerns and deriving patient-specific cells has enhanced their potential in regenerative medicine, drug screening, and disease modeling (Takahashi et al., 2007). Over the past decade, these cells have been successfully differentiated into different cell types, including neural progenitors (Chambers et al., 2009), cardiomyocytes (Lian et al., 2014), and liver buds (Takebe et al., 2017).

In addition, the need for large numbers of cells for drug screening and cell therapies has led to the development of efficient and reproducible platforms for the expansion and directed differentiation of hPSCs. Lately, this need has been addressed in a three-dimensional (3D) perspective, which aims to mimic *in vivo* cell-cell and cell-extracellular matrix interactions. The 3D model format also enables other advantages, as demonstrated in the derivation of cardiomyocytes from hiPSCs, which present a higher level of maturation under 3D conditions compared to adherent approaches (Branco et al., 2019).

Although different culture media formulations for the expansion of hPSCs have been developed, most were formulated for culture under adherent conditions, either static or dynamic. When considering the 3D expansion of hiPSCs, using a suspension culture-specific medium such as Gibco StemScale PSC Suspension Medium (StemScale) is highly advantageous. StemScale will promote higher cell aggregation from single-cell populations and enable better aggregate expansion potential, as it is specifically adapted to promote cell growth under suspension conditions.

One of the main constraints in 3D culture relies on the diffusional gradients of nutrients, metabolites, and gases such as oxygen and carbon dioxide. Improper diffusion of these gases and metabolites in 3D culture can potentially cause cell death or differentiation. The use of agitated conditions, including the use of vertical-wheel bioreactors, can reduce these limitations while simultaneously maintaining homogeneous aggregate diameters (Wu et al., 2014; Dang et al., 2021). These types of bioreactors have already been applied toward hiPSC expansion (Nogueira et al., 2019; Nogueira et al., 2021) and directed differentiation of hiPSC into the cerebellum (Silva et al., 2020b).

In fact, recent advances in cell culture media technology enabled simplified and efficient protocols for neural induction of hiPSCs. Traditional approaches toward neural induction include dual SMAD signaling inhibition (Chambers et al., 2009) and are based on the manual addition of small molecule inhibitors, such as SB431542 and LDN-193189, which can lead to some variability in the efficiency of the protocol. The PSC Neural Induction Medium is a defined serum-free media that allows a rapid generation of neural progenitors in only 7 days under adherent conditions (Yan et al., 2013). The use of the PSC Dopaminergic Neuron Differentiation Kit can shorten the period of midbrain floor plate progenitor generation to 6 days, and the complete differentiation period of dopaminergic neurons to only 25 days.

In this work, we were able to scale up the expansion of hiPSCs using a newly developed culture medium designed for suspension conditions and capable of generating up to 665-fold change in hiPSC cell numbers under dynamic conditions in only 15 days. Furthermore, neural induction of hiPSC in spinner flasks using the PSC Neural Induction Medium yielded up to 30-fold change in cell number in 7 days. Finally, it was possible to develop three neural induction protocols that yield lineage-specific progenitors, including hindbrain and midbrain floor plate progenitors.

## Materials and Methods

### Maintenance of Human Induced Pluripotent Stem Cells

Human induced pluripotent stem cells, Gibco Human Episomal iPSC line derived from CD34 + cord blood (iPSC6.2, GibcoEpi (Burridge et al., 2011)) and F002.1A.13 (TCLab – Tecnologias Celulares para Aplicação Médica, Unipessoal, Lda., Portugal) were routinely cultured on Matrigel (1:100, Corning)-coated plates using StemFlex medium (Gibco). Cells were passaged 1:5 using 0.5 mM EDTA (Life Technologies) every 5 days (Beers et al., 2012).

### Cell Expansion Under Agitated Conditions

A single-cell suspension of hiPSCs was obtained by incubation with Accutase (Sigma) at 37°C for 5 min. Cells were seeded at 1.5 × 10^5^ cells/mL using StemScale PSC Suspension Medium (StemScale, Gibco) supplemented with 10 μM ROCK inhibitor Y-27632 (StemGent) for the first 24 h. 50% of the medium volume was replaced every-other-day. Cells were cultured either on a 6-well ultra-low attachment plate (Corning) cultured on top of an orbital shaker platform (Rotamax 120, Heidolph) or in PBS Mini 0.1 bioreactors (PBS Biotech), following established protocols (Miranda et al., 2016; Nogueira et al., 2019). Cells were recovered after 3–5 days after seeding, dissociated for cell counting, viability assessment and re-seeded at 1.5 × 10^5^ cells/mL. This process was repeated three times for each system (P1, P2, P3) and, at P3, cells were recovered and replated at 1 × 10^5^ cells/cm^2^ for immunostaining or stored for further analysis.

### Neural Induction of Human Induced Pluripotent Stem Cells

For midbrain specification, the PSC Dopaminergic Neuron Differentiation kit (Gibco) was used, comprising a period of 6 days culturing cells in Floor Plate (FP) Specification Medium and 5 days in FP Expansion Medium. During this phase, cells were split 1:2 or 1:3 without aggregate dissociation at day 6 and at day 9, and the excess cells were discarded. Further maturation was achieved using Dopaminergic Neuron Maturation Medium, according to manufacturer instructions. For general neural differentiation, hiPSC were cultured for 7 days using PSC Neural Induction Medium (NIM, Gibco). For hindbrain specification, hiPSC were cultured for 6 days in NIM, which was supplemented with 1 μM retinoic acid from day 3 to day 6. The culture medium was replaced on day 6, using Neural Expansion Medium (NEM) up to day 11. Cells were cultured in spinner flasks using previously described conditions (Miranda et al., 2016). Neuronal maturation of cells up to day 34 of differentiation was pursed according to a previously described protocol, using NEM or N2B27 without supplementation (Miranda et al., 2020).

### LIVE/DEAD Assay

To assess cell viability after expansion or neural induction of hiPSCs, a 0.5 mL cell suspension sample was transferred into an ultra-low attachment 24-well plate. Cells were treated with 0.1 μM Calcein-AM and 8 μM Ethidium homodimer-1 (LIVE/DEAD^®^ Viability/Cytotoxicity Kit for mammalian cells, Invitrogen) at room temperature for 20 min and then observed under a fluorescence optical microscope and a digital camera.

### Immunostaining

Cells were fixed with 4% PFA and stained according to a previously described protocol (Miranda et al., 2018). Primary and secondary antibodies are listed on Supplementary Table S1. Fluorescence images were acquired either with the fluorescence optical microscope (Leica DMI 3000B) and a digital camera (Nikon DXM 1200), or with Zeiss LSM 710 Confocal Laser Point-Scanning Microscope using 20x objective with 80% digital zoom, and integrated density was calculated for each channel using ImageJ software.

### Aggregate Size Measurement and Analysis

To monitor aggregate sizes over time in culture, photographs were taken using a Leica DMI 3000B microscope with a Nikon DXM 1200F digital camera. Using ImageJ, the area of each aggregate was determined, and the respective diameter was calculated. Considering the imaged aggregates as spheroids, the diameter was calculated according to the equation d = 2⋅√(A/π), in which d represents the diameter and A represents the area. The diameter value was then converted from pixels to μm, using a conversion factor empirically determined with the scale bar as a reference.

### Quantitative Real-Time-PCR

For quantitative analysis, total RNA was extracted at different time points of differentiation and treated using High Pure RNA Isolation Kit (Roche), according to the manufacturer’s instructions. Total RNA was converted into complementary cDNA with a High-Capacity cDNA Reverse Transcription Kit (Thermo Fisher Scientific) using 100 μg of RNA. Relative gene expression was evaluated using 50 μg of cDNA and 250 μM of each primer. Expression levels were analyzed using SYBR^®^ green chemistry (Nzytech), with primers for *PAX6*, *NESTIN*, *SOX2*, *PAX2*, *EN2*, *OTX2*, *GBX2*, *TH*, and *Brachyury (T)* (Supplementary Table S2). All PCR reactions were done in triplicate, using the StepOne*™* Real-Time PCR System (Thermo Fisher Scientific). Quantification was performed by calculating the ΔCt value using GAPDH as a reference gene and results are shown as mRNA expression levels (2^–ΔCt^) relative to GAPDH. Log2 normalized expression values of the average fold-change were used for ClustVis analysis of neural genes (Metsalu and Vilo, 2015).

### Single-Cell Calcium Imaging

Intracellular variations of Ca^2+^ were analyzed by single-cell calcium imaging (SCCI). Cells were re-plated on Glass Bottom Cell Culture Dish (Nest) previously coated with poly-L-ornithine (15 μg/mL, Sigma) and Laminin (20 μg/mL, Sigma). Calcium indicator Fura-2, a fluorescent dye that switches its excitation peak from 340 to 380 nm when bound to calcium, allows the concentration of intracellular calcium to be determined based on the ratio of fluorescence emission after sequential excitation at 340 and 380 nm (Grienberger and Konnerth, 2012). Cells were preloaded with 5 μM Fura-2 AM (Invitrogen) in Krebs solution (132 mM NaCl, 4 mM KCl, 1.4 mM MgCl_2_, 2.5 mM CaCl_2_, 6 mM glucose, 10 mM HEPES, pH 7.4) for 45 min at 37°C in an incubator with 5% CO2 and 95% atmospheric air. Dishes were washed in Krebs solution and then mounted on an inverted microscope with epifluorescence optics (Axiovert 135TV, Zeiss). Cells were stimulated by applying high-potassium Krebs solution (containing 10–100 mM KCl, isosmotic substitution with NaCl), or 100 μM histamine. Ratio images were obtained from image pairs acquired every 200 ms by exciting the cells at 340 nm and 380 nm. Excitation wavelengths were changed through a high-speed switcher (Lambda DG4, Sutter Instrument, Novato, CA, United States). The emission fluorescence was recorded at 510 nm by a cooled CDD camera (Photometrics CoolSNAP fx). Images were processed and analyzed using the software MetaFluor (Universal Imaging, West Chester, PA, United States). Regions of interest were defined manually.

### PluriTest and KaryoStat Characterization

To characterize the cell populations after expansion, single cells obtained from dissociated aggregates were frozen as cell pellets and sent for analysis using the PluriTest Assay (Thermo Fisher Scientific) and KaryoStat Assay (Thermo Fisher Scientific). PluriTest characterization compares the transcriptome of the submitted sample against an extensive reference set, to determine how well the samples have maintained pluripotency. KaryoStat characterization is an alternative to G-band karyotyping, which analyzes the entire sample genome and reports any potential chromosomal aberrations that may be detected (Kamand et al., 2020; Kamand et al., 2021).

### Heatmaps

ClustVis analysis software^1^ was used for hierarchical clustering, with the rows clustered using correlation distance and average linkage (Metsalu and Vilo, 2015).

### Statistical Analysis

Error bars represent the standard error of the mean (SEM). Unless otherwise stated, at least three independent experiments were run for each set of data. When appropriate, statistical analysis was performed using a two-tailed Student’s *t*-test for independent samples, and a *p*-value less than 0.05 was considered statistically significant.

## Results

### StemScale Promotes Efficient Human Induced Pluripotent Stem Cell Expansion Under Agitated Conditions

**FIGURE 1:**
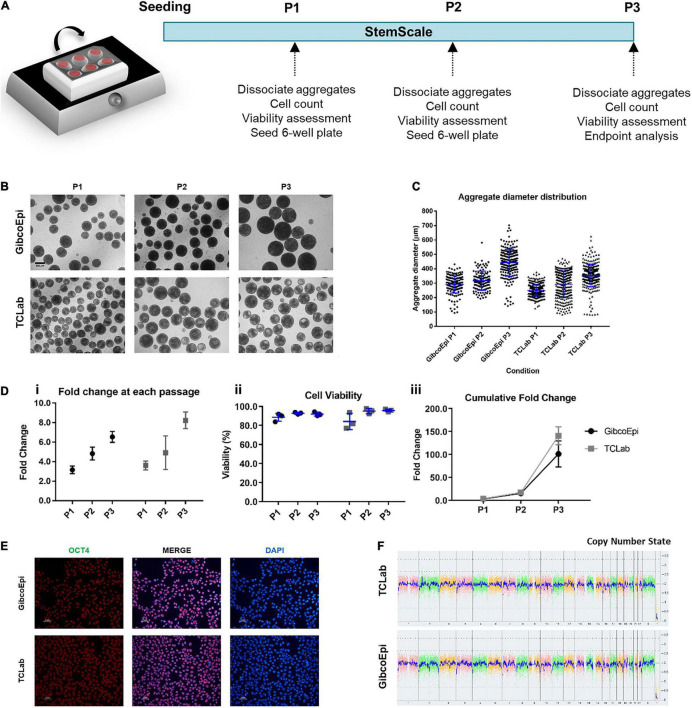
Expansion of hiPSCs under agitated conditions using an orbital shaker. **(A)** Schematic overview of hiPSC expansion on orbital shaker using StemScale. **(B)** Bright Field images of 3D aggregates before P1, P2 and P3 for GibcoEpi and TCLab cells lines. Scale bars: 300 μm. **(C)** Distribution of hiPSC aggregate diameters obtained using StemScale before P1, P2 and P3. **(D)** Fold change **(i)**, cell viability **(ii)** and cumulative fold change **(iii)** of hiPSC at P1, P2 and P3. **(E)** Immunocytochemical analysis of hiPSC obtained after three passages on orbital shaker and 48 h days after replating in Matrigel-coated plates for Oct4 expression. Nuclei were stained with DAPI. Scale bars: 50 μm. **(F)** At the end of three passages, the karyotypic analysis remained normal.

Assessment of cell growth under agitated conditions, namely on an orbital shaker platform, was performed through culturing hiPSCs over three consecutive passages (Figure 1A). For this, cells were seeded at a density of 1.5 × 10^5^cells/mL in 6-well ultra-low attachment plates, using StemScale supplemented with 10 μM Y-27632. The plates were immediately transferred to an orbital shaker platform set to an agitation rate of 70 RPM. 50% of StemScale culture medium was replaced daily, and cells were cultured up to 5 days, depending on the average aggregate diameter. When aggregates were ready to passage and images had been acquired (Figure 1B), the aggregates were dissociated using Accutase and the fold change and viability were assessed. Recovered cells were seeded at the same cell density in new 6-well ultra-low attachment plates, and cultured up to 5 days, repeating the same protocol until a total of three passages were performed. The average diameter of the aggregates for each passage was 291 ± 59 μm, 317 ± 66 μm, and 442 ± 94 μm, for P1 (3 days), P2 (4 days), and P3 (5 days), respectively, for the GibcoEpi cell line, and averaging 249 ± 37 μm, 292 ± 78 μm, and 352 ± 78 μm, for P1 (4 days), P2 (4 days), and P3 (5 days), respectively, for the TCLab cell line (Figure 1C). Cell growth during these periods resulted in a fold change of 3.2 ± 0.2-fold (P1), 4.8 ± 0.4-fold (P2), and 6.5 ± 0.3-fold (P3) for the GibcoEpi cell line (Figure 1Di), with a percentage of cell viability higher than 88% for every passage (Figure 1Dii), and a cumulative fold change of 102 ± 16-fold after 3 passages (Figure 1Diii). In the case of the TCLab cell line, cell growth achieved a fold change of 3.6 ± 0.3 (P1), 4.9 ± 1.0 (P2), and 8.2 ± 0.5 (P3) during each passage (Figure 1Di), resulting in a cumulative fold change of 141 ± 11 (Figure 1Diii), while maintaining cell viability higher than 84% (Figure 1Dii).

Cells were recovered at the end of the three passages and, upon replating, were tested for the pluripotency marker OCT4 (Figure 1E), and image analysis revealed a percentage of OCT4-positive cells of 90.4 ± 2.8% and 91.6 ± 2.8% for GibcoEpi and TCLab, respectively. Recovered cells were also analyzed using an algorithm that integrates bioinformatics to authenticate pluripotency potential, which was confirmed (Supplementary Table S3). Moreover, no chromosomal aberrations in both cell lines were found when comparing against the reference dataset (Figure 1F).

### Scale-Up Using PBS Bioreactors Yields Large Numbers of Human Induced Pluripotent Stem Cells

**FIGURE 2:**
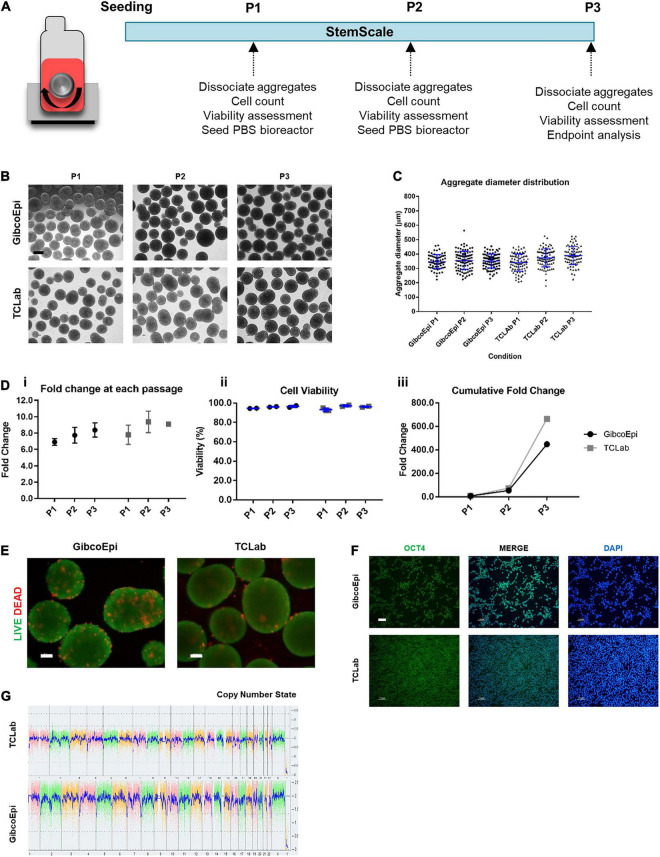
Expansion of hiPSCs under agitated conditions using vertical-wheel bioreactors. **(A)** Schematic overview of hiPSC expansion on vertical-wheel bioreactor using StemScale. **(B)** Bright Field images of 3D aggregates before P1, P2, and P3 for GibcoEpi and TCLab cells lines. Scale bars: 100 μm. **(C)** Distribution of hiPSC aggregate diameters obtained using StemScale before P1, P2, and P3. **(D)** Fold change **(i)**, cell viability **(ii)** and cumulative fold change **(iii)** of hiPSC at P1, P2, and P3. **(E)** Viability of cells in 3D aggregates at P3, using LIVE/DEAD staining kit. Green: calcein-AM; Red: Ethidium homodimer-1. Scale bars: 100 μm. **(F)** Immunocytochemical analysis of hiPSC obtained after three passages on vertical-wheel bioreactor and 48 h days after replating in Matrigel-coated plates for Oct4 expression. Nuclei were stained with DAPI. Scale bars: 100 μm. **(G)** At the end of three passages, the karyotypic analysis remained normal.

Following the initial approach to sustain hiPSC expansion using StemScale, a scale-up method was pursued by using PBS 0.1 Mini Bioreactors, as described in Figure 2A. For this, the two hiPSC lines were seeded in 60 mL of StemScale supplemented with 10 μM Y-27632, using the same cell density of 1.5 × 10^5^ cells/mL and an agitation rate of 40 RPM. At day 1, 40 mL of StemScale was added to the PBS 0.1 Mini Bioreactor (resulting in a final culture volume of 100 mL), followed by a 50% medium exchange every-other-day until day 5 of expansion. After 5 days of culture, hiPSC aggregates were dissociated and seeded at a density of 1.5 × 10^5^ cells/mL in a new PBS 0.1 Mini Bioreactor. Cells were passaged every 5 days for a total of three passages and, during each passage, hiPSC aggregate morphology was analyzed. Bright-field images of the aggregates show no agglomeration of aggregates (Figure 2B). The diameter of hiPSC aggregates was also analyzed (Figure 2C), averaging 347 ± 48 μm, 355 ± 64 μm, and 351 ± 48 μm, for P1, P2, and P3, respectively, for the GibcoEpi cell line, and averaging 340 ± 60 μm, 369 ± 75 μm, and 389 ± 63 μm, for P1, P2, and P3, respectively, for the TCLab cell line. Overall, the aggregate diameter distribution was similar between passages and cell lines, and the average diameter for every condition was below 400 μm.

Fold change in cell number, cell viability, and cumulative fold change were assessed at each passage. Considering the cell growth at each passage, the GibcoEpi cell line was able to sustain a fold change of 6.9 ± 0.3 at P1, 7.7 ± 0.7 at P2, and 8.2 ± 0.6 at P3, while the TCLab cell line sustained a fold change of 7.8 ± 0.7 at P1, 9.4 ± 0.9 at P2, and 9.1 ± 0.1 at P3 (Figure 2Di). In addition, cell viability was continuously maintained above 93% (Figure 2Dii). Considering the three passages, over 15 days of expansion it was possible to obtain a cumulative fold change of 448-fold and 665-fold for GibcoEpi and TCLab cell lines, respectively (Figure 2Diii). Potential necrotic zones within the aggregates at the end of P3 were determined to not have developed, confirmed by absence of the localized presence of Et-1-h. Calcium-AM staining demonstrates that most cells within the aggregates remain viable (Figure 2E). After 15 days of expansion, aggregates were dissociated and replated in Matrigel-coated plates. 24 h after replating, cells were stained and tested positive for the presence of the pluripotency marker OCT4 (Figure 2F). Using the KaryoStat Assay, cells expanded for 15 days in PBS 0.1 Mini Bioreactors were tested for karyotypic changes, and no chromosomal aberrations were found when comparing against the reference dataset (Figure 2G).

### Scaling Up the Production of Human Induced Pluripotent Stem Cell-Derived Neural Progenitors Using PSC Neural Induction Medium

**FIGURE 3:**
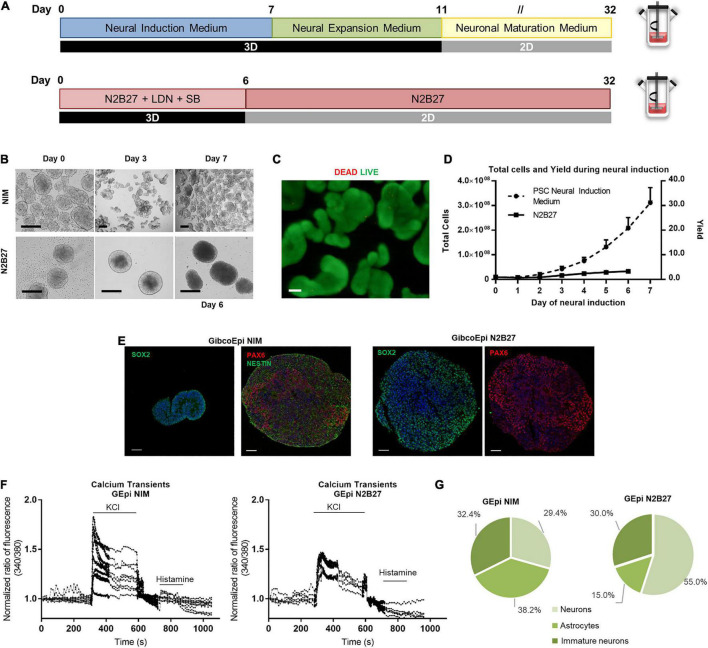
Efficient and scalable generation of neural progenitors using Neural Induction Medium in spinner flasks. **(A)** Schematic overview of neural induction of hiPSC on spinner flasks using Neural Induction Medium (NIM) and N2B27. **(B)** Bright field images of aggregates at day 0, 3, and 7 of neural induction. Scale bars: 300 μm. **(C)** Viability of cells in 3D aggregates after hiPSC neural induction (NIM), using LIVE/DEAD staining kit. Green: calcein-AM; Red: Ethidium homodimer-1. Scale bars: 100 μm. **(D)** Growth curves of hiPSC cultured in spinner flasks through 7 days of neural induction, using NIM or N2B27, for total cell number and cell yield. **(E)** Immunocytochemical analysis of hiPSC-derived neural progenitor aggregates obtained after neural induction in spinner flasks for Nestin, Pax6 and Sox2 expression. Nuclei were stained with DAPI. Scale bars: 50 μm. **(F)** Single-cell calcium imaging (SCCI) analysis at day 34 of neural induction. **(G)** Percentage of neurons, immature neurons and glial cells calculated based on the response to KCl and histamine.

The workflow for the neural induction of hiPSC in spinner flasks is shown in Figure 3A. Briefly, using previously optimized parameters regarding working volumes and agitation rates (Miranda et al., 2016), 125-mL spinner flasks were inoculated with hiPSC at a density of 2.5 × 10^5^ cells/mL to generate hiPSC aggregates ranging from 150 to 200 μm in diameter. After 24 h, the culture medium was replaced by PSC Neural Induction Medium or N2B27 supplemented with 10 μM SB431542 and 100 nM LDN193189 for 7 or 6 days, respectively.

The first difference is observable in terms of aggregate morphology (Figure 3B). Whereas N2B27 aggregates display a larger size, NIM aggregates tend to reach a smaller size, and different aggregates start to stem from the original one. Cell viability and absence of necrotic areas in the NIM aggregates were verified by LIVE/DEAD staining after the neural induction protocol inside the spinner flasks (Figure 3C). The average growth curve obtained during the neural induction period is depicted in Figure 3D. A maximum of (3.3 ± 0.6) × 10^6^ cells and (3.1 ± 0.6) × 10^6^ cells were obtained at days 6 and 7 after starting the neural induction with the N2B27 and NIM media, respectively, which corresponds to a fold change in cell number of 3.4 ± 0.5-fold for N2B27 and 31.1 ± 6.2-fold for NIM. On day 6 (N2B27) or day 7 (NIM), aggregates were recovered and cut into sections for further analysis. There was positive staining for neural markers in both conditions, including SOX2, NESTIN, and PAX6 (Figure 3E).

Recovered aggregates were also replated into poly-ornithine/laminin-coated plates and cells were matured up to day 34 of differentiation, either using N2B27 or NEM. At day 34, single-cell calcium imaging (SSCI) was performed to further evaluate the functionality of the cells, as well as neuron-glia ratio. Upon KCl stimulation, a sharp increase in cytosolic calcium concentration of 1.8-fold on average was observed in cells induced using NIM, whereas cells cultured in N2B27 displayed a maximum of 1.45-fold change (Figure 3F). Moreover, the ratio between histamine and KCl responses can be used to evaluate the proportion of different cell populations, including neurons, astrocytes and immature neurons (Agasse et al., 2008). In fact, cells undergoing a differentiation protocol using NIM yielded similar proportions of the three cell types, whereas N2B27 promoted 55.0% of neurons, 15.0% astrocytes, and 30% of immature neurons (Figure 3G and Supplementary Figure S1).

### Scaling Up the Production of Human Induced Pluripotent Stem Cell-Derived Floor Plate Progenitors Using PSC Dopaminergic Neuron Differentiation Kit

**FIGURE 4:**
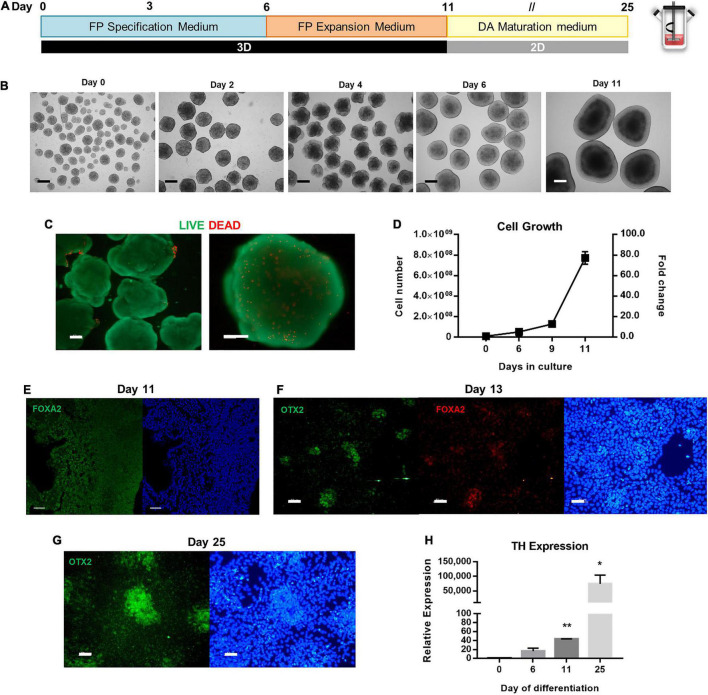
Scalable differentiation of hiPSCs into FP Progenitors using spinner flasks. **(A)** Schematic overview of FP specification and dopaminergic neuron maturation until day 25 of differentiation. **(B)** Bright Field images of 3D aggregates between day 0 and day 11. Scale bars: 300 μm. **(C)** LIVE/DEAD assay of FP progenitors recovered at day 11. Green: calcein-AM; Red: Ethidium homodimer-1. Scale bars: 100 μm. **(D)** Growth curves of hiPSC undergoing FP specification and expansion in spinner flasks through 11 days, for total cell number and cell yield. Representative immunofluorescence images for the FP marker FoxA2 in aggregates recovered at day 11 **(E)**, on replated cells at day 13 **(F)**, and Otx2 on replated cells at day 25 **(G)**. Scale bars: 50 μm. **(H)** Quantitative RT-PCR analysis of TH mRNA expression levels (2^–ΔΔCt^) relative to GAPDH. *p < 0.05; **p < 0.001.

For the derivation of floor plate progenitors in spinner flasks, seeding conditions were adapted from the previous protocol (Figure 4A). To generate hPSC aggregates with an average diameter of 120 to 180 μm for the first 24 h after inoculation, the agitation rate was set to 40 RPM. During the neural induction protocol, the agitation rate was maintained at 45 RPM. Cells were cultured under 3D conditions for 6 days in FP Specification Medium followed by 5 days of culture in FP Expansion Medium. During this period, cells were split at day 6 and day 9 to ensure sufficient nutrient feeding.

The morphology of the aggregates was assessed throughout the differentiation process (Figure 4B). Aggregates acquire a “bubbly” morphology at day 2, which disappears by day 6. At day 11, it is possible to distinguish apico-basally polarized-like tissue in the aggregates, which reach diameters up to 1 mm. Despite the large size of the aggregates, no necrotic zones were identified through the LIVE/DEAD assay, and most cells remain viable (Figure 4C). In terms of cell number, when comparing the PSC Dopaminergic Neuron Differentiation Kit with N2B27, or even with NIM, this protocol generates the highest cell numbers starting with the same number of cells seeded in the spinner flask (Figure 4D). The fold change in cell number averages 5.1 ± 0.3-fold on day 6, 12.8 ± 3.1-fold on day 9, and 77.2 ± 6.1-fold on day 12. A linear regression on the growth curve yielded a specific growth rate of 0.36 d^–1^ (*R*^2^ = 0.90) and a doubling time of 45.7 h.

Analysis of the aggregates recovered at day 11 demonstrates the presence of the floor plate marker FOXA2 (Figure 4E), which is maintained after the aggregates are replated into Matrigel-coated plates and co-localized with OTX2 (Figure 4F), and by day 25 there are also present some clusters of cells that express OTX2 (Figure 4G). Quantification of TH, a dopaminergic neuron marker, was performed through quantitative real-time polymerase chain reaction (qRT-PCR) analysis, before and after induction, as well as during the dopaminergic maturation period (Figure 4H). Expression of TH dopaminergic neuron gene is upregulated throughout the neuronal maturation, with the maximum value of relative expression at day 25.

### Differences in Gene Expression With Different Differentiation Protocols

**FIGURE 5:**
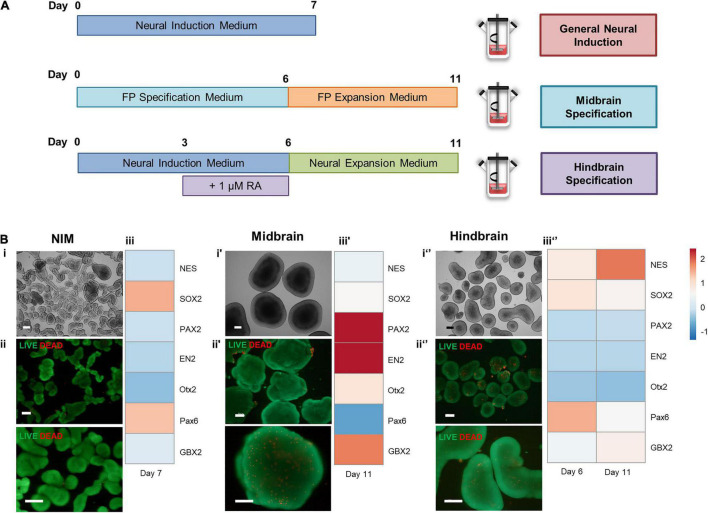
**(A)** Schematic overview of different approaches to induce hiPSCs in spinner flasks. **(B)** Cell characterization after neural induction of hiPSCs in spinner flasks. Bright field images of 3D aggregates recovered at day 7 **(i′)** and day 11 **(i,i″).** LIVE/DEAD assay of FP progenitors recovered at day 7 **(ii′)** or day 11 **(ii,ii″)**. Green: calcein-AM; Red: Ethidium homodimer-1. Scale bars: 200 μm. Hierarchical clustering illustrates relative expression levels of different genes for hiPSC and hiPSC-derived neural progenitors **(iii,iii′,iii″)**. *NES*, *SOX2, PAX2, EN2, OTX2, PAX6*, and *OTX2* were analyzed by qRT-PCR.

To address the possibility of generating different neural regions from hiPSCs at a spinner flask scale, different approaches to induce hiPSCs were applied (Figure 5A). Neural induction using NIM alone generated smaller aggregates (Figure 5Bi), comprised mostly with viable cells (Figure 5Bii), and yielded upregulation of SOX2 and PAX6 (Figure 5Biii). To generate midbrain progenitors, a protocol of 6 days of FP specification and 5 days of FP progenitor expression was employed, and hindbrain specification was pursued by supplementing NIM with 1 μM of retinoic acid. Hindbrain progenitors were also expanded by using a neural expansion medium for five extra days. Midbrain specification generated large aggregates with apicobasal-like tissue on the outer layer (Figure 5Bi′), and no necrotic zones were identified within the aggregates (Figure 5Bii′). Gene expression analysis through qRT-PCR revealed upregulation of midbrain markers, including PAX2 and EN2, and OTX2 (Figure 5Biii′), compared to a hiPSC control. Hindbrain specification using a combination of NIM and retinoic acid-derived medium-sized aggregates (Figure 5Bi″), without the presence of necrotic zones (Figure 5Bii″). The addition of retinoic acid was able to caudalize the aggregate population comparing to the use of NIM alone, as seen by the upregulation of the GBX2 hindbrain gene (Figure 5Biii″). Additionally, there was no upregulation of the mesodermal marker T/Brachyury in none of the differentiation protocols tested (Supplementary Figure S2).

## Discussion

The use of hiPSCs in clinical settings for drug discovery or even cell therapy will require the production of large numbers of hiPSCs and their derivatives. To accomplish this, robust protocols that allow the production of high-quality cells are currently being developed. Here, we demonstrated two strategies for expanding hiPSCs as suspension aggregates, either in orbital shakers or vertical-wheel bioreactors.

The first strategy comprises the use of an orbital shaker that enhances the homogeneity of nutrients, metabolites and gases in the culture media, while promoting a more uniform aggregation of hiPSCs that enhances cell aggregation and aggregate homogeneity (Miranda et al., 2015). Expansion of hiPSCs on the orbital shaker platform demonstrated that the difference in timing of the passage leads to differences in the fold change of hiPSC number and the diameter of the aggregates. Nevertheless, both hiPSC lines used in this study were able to maintain high viability throughout the time of culture, and cell growth using StemScale with orbital shaker platform cultures can be compared to previously obtained performance (Miranda et al., 2016).

Single-cell suspensions of hiPSCs depend on the addition of a ROCK inhibitor to promote cell survival (Watanabe et al., 2007), and although agitated conditions enhance cell aggregation, some advances postulate that the addition of polysulfated compounds may prevent hiPSC aggregate agglomeration while promoting single-cell aggregation (Lipsitz et al., 2018). One of the main constraints often associated with suspension cultures is the agglomeration of several cell aggregates, which may lead to culture heterogeneity and diminished diffusion of gases, nutrients, and metabolites, which may ultimately lead to localized cell death (Kinney et al., 2011). In this work, StemScale alone was sufficient to promote hiPSC aggregation using only Y-27632 supplementation and, at the same time, providing a flexible feeding schedule. The diameters of the hiPSC aggregates do not exceed 400 μm, and necrotic zones within the center of the aggregates were not detected by calcein-AM/ethidium homodimer staining and microscopy analysis after three consecutive passages in the bioreactor.

Despite the expansion potential of 3D hiPSC suspension culture, the need for large numbers of cells for the potential use of hiPSC derivatives in clinical applications relies on their scalability. However, these systems often compromise cell viability due to shear stress that tends to increase in agitated conditions. This issue can be surpassed by using vertical-wheel bioreactors, designed to offer homogeneous culture conditions and, importantly, to provide a gentler agitation mechanism (Croughan et al., 2016). The fold change in cell number obtained with this platform that combines the use of vertical-wheel bioreactors and StemScale demonstrates a high scalability potential. Here, we developed a platform for hiPSC expansion under suspension conditions that yield up to a cumulative 665-fold change in cell number in only 15 days. Considering just 5 days of expansion, which corresponds to one passage, StemScale can yield up to a 9.3-fold change in cell number, which could only be attained with other hPSC culture media in 7 days (Nogueira et al., 2019). The use of other culture media formulations in the same vessels, such as mTeSR1, has yielded a maximum fold change in cell number of 4.8-fold in 7 days, whereas the addition of dextran sulfate, a polysulfated compound that reduces cells aggregation and modulates charges in the surface of cells (Lipsitz et al., 2018), doubled this number (Nogueira et al., 2019). The use of fully-controlled perfusion systems for hiPSC expansion has recently been demonstrated to yield hiPSC density up to 35 million cells/mL (Manstein et al., 2021), and the scalability potential of StemScale culture medium could be tested using other vertical-wheel configurations, such as the PBS 3L. In hPSC *in vitro* culture, it is important to control the karyotype of the cells, as chromosomal aberrations are more prone to arise in cells with high expandability. This is especially relevant in hPSC expansion, where we aim to obtain high fold changes in cell numbers. StemScale has demonstrated its ability to maintain a normal karyotype in two different hiPSC lines, either after expansion on orbital shakers or in vertical-wheel bioreactors.

The recapitulation of developmental processes *in vitro* heavily relies on the control over the initial aggregate diameter to attain optimal differentiation efficiencies (Bauwens et al., 2008; Miranda et al., 2018). Scale-up strategies using a dual SMAD inhibition protocol have been demonstrated to yield a fold change averaging 3.0-fold in 6 days neural of induction (Miranda et al., 2016). The use of NIM enhances this potential, either by raising the fold change by almost 10-fold in only 7 days, or by originating aggregates that already display some level of structural organization. Upon further maturation of the progenitors, it was possible to determine that were present glial cells, neurons, and immature neurons that can still differentiated. The determined ratio between these three types of cells is similar in terms of overall percentage, which matches previous results (Yan et al., 2013).

Nevertheless, to specific applications that may require a smaller percentage of glial cells, adding a specific cell culture supplement – Culture One Supplement – could decrease the number of glial cells. Furthermore, the use of agitated conditions has demonstrated the ability to accelerate hiPSC differentiation into neural lineages. The transcriptomic profile from cerebellar organoids derived in vertical-wheel bioreactors suggests an enhanced functional maturation, which can be explained by an enrichment of extracellular matrix components that allow a better mimicking of the 3D neural microenvironment (Silva et al., 2021).

Since hPSCs have an unlimited potential to generate different cell types, we next wanted to assess the scalability of the production of midbrain floor plate progenitors from hiPSCs. The floor plate progenitor aggregates display a different morphology than the aggregates generated using the NIM, maintaining a structural organization within bigger-sized aggregates. In terms of cell number, although cells were passaged and split during the expansion of the floor plate progenitors, if we were considering the inoculation of different parallel spinner flasks, we could potentially obtain up to an 80-fold change in just 11 days. These progenitors were also positive for FOXA2 and OTX2 expression (Figure 4F) and, although OTX2 alone is also present in the cerebellum, the presence of FOXA2 is a common marker for floor plate progenitors (Xi et al., 2012). The expression of TH, a dopaminergic neuron marker, increases throughout the days of dopaminergic induction, which is consistent with the maturation of floor plate progenitors into dopaminergic neurons (Xi et al., 2012).

To our knowledge, this is the first scalable suspension 3D approach to derive midbrain floor plate progenitors from hiPSCs. Nevertheless, in order for these cells to be used for clinical applications, the ability of *in vivo* engraftment of the derived dopaminergic neurons remains to be assessed (Kriks et al., 2011), as well as the lack of generation of teratomas upon transplantation.

The possibility of generating different types of brain regions from hiPSC can facilitate their translation into several applications, such as drug testing and disease modeling (Miranda et al., 2018; Miranda et al., 2020; Silva et al., 2020a). The utilization of NIM has been shown to enable the derivation of neural progenitors in a more primitive state, which facilitates the derivation of different brain regions (Yan et al., 2013). We introduced a caudalizing factor, retinoic acid, to promote shifting toward a hindbrain identity to evaluate this hypothesis. As expected, we were able to generate hindbrain progenitors that exhibited upregulation of characteristic genes, such as GBX2 (Kirkeby et al., 2012), compared to cells derived using NIM without any further supplementation. Although retinoic acid has been shown to suppress differentiation of ventral CNS tissues and promote the induction of the caudal genes, it acts in a dose- and time-dependent manner (Irioka et al., 2005). Therefore, in our studies we maintained the cells in the presence of retinoid acid for a short period and low concentration to avoid inducing cells to a spinal cord identity (Irioka et al., 2005).

## Data Availability Statement

The original contributions presented in the study are included in the article/Supplementary Material, further inquiries can be directed to the corresponding author.

## Author Contributions

CM, MA, MD, MV, JC, and TF conceived and designed the experiments. CM and TS performed the experiments. CM, MA, TS, MD, MV, JC, and TF analyzed the data. MA, MD, MV, JC, and TF contributed reagents, materials, and analysis tools. CM, MA, MV, JC, and TF wrote the manuscript. All authors contributed to the article and approved the submitted version.

## Conflict of Interest

MA, MD, and MV were employed by Thermo Fisher Scientific and receive financial compensation as salaried employees. The remaining authors declare that the research was conducted in the absence of any commercial or financial relationships that could be construed as a potential conflict of interest.

## Publisher’s Note

All claims expressed in this article are solely those of the authors and do not necessarily represent those of their affiliated organizations, or those of the publisher, the editors and the reviewers. Any product that may be evaluated in this article, or claim that may be made by its manufacturer, is not guaranteed or endorsed by the publisher.

## Footnotes

1
http://biit.cs.ut.ee/clustvis
