# Supplementary material for: A Dynamic 3D Aggregate-Based System for the Successful Expansion and Neural Induction of Human Pluripotent Stem Cells
Source: Front Cell Neurosci. 2022 Mar 3;16:838217. doi: 10.3389/fncel.2022.838217 (PMC8928726; doi:10.3389/fncel.2022.838217)
Supplement: Supplementary file 1 [file Data_Sheet_1.docx]

**Supplementary Information**

**A Dynamic 3D Aggregate-Based System for the Successful Expansion and Neural Induction of Human Pluripotent Stem Cells**

Cláudia C. Miranda, Michael L. Akenhead, Teresa P. Silva, Michael A. Derr, Mohan C. Vemuri, Joaquim M. S. Cabral, Tiago G. Fernandes

**Table S1** Primary antibody dilutions used for immunostaining.

| **Antibody** | **Brand** | **Host species** | **Dilution** |
| --- | --- | --- | --- |
| OCT4 | Millipore | Mouse | 1:750 |
| SOX2 | R&D Systems | Mouse | 1:200 |
| PAX6 | Covance | Rabbit | 1:400 |
| NESTIN | R&D Systems | Mouse | 1:400 |
| FOXA2 | R&D Systems | Goat | 1:40 |
| OTX2 | Millipore | Rabbit | 1:400 |

**Table S2** Primers used for qRT-PCR.

| **Gene** | **Sequence** | |
| --- | --- | --- |
| *GAPDH* | Forward | GAGTCAACGGATTTGGTCGT |
|  | Reverse | TTGATTTTGGAGGGATCTCG |
| *SOX2* | Forward | GGGAAATGGGAGGGGTGCAAAAGAGG |
|  | Reverse | TTGCGTGAGTGTGGATGGGATTGGTG |
| *NESTIN* | Forward | GAAACAGCCATAGAGGGCAAA |
|  | Reverse | TGGTTTTCCAGAGTCTTCAGTGA |
| *PAX6* | Forward | GAATCAGAGAAGACAGGCCA |
|  | Reverse | GTGTAGGTATCATAACTCCG |
| *PAX2* | Forward | AACGACAGAACCCGACTATGT |
|  | Reverse | GAGCGAGGAATCCCCAGGA |
| *OTX2* | Forward | AGAGGACGACGTTCACTCG |
|  | Reverse | TCGGGCAAGTTGATTTTCAGT |
| *EN2* | Forward | CCGGCGTGGGTCTACTGTA |
|  | Reverse | GGCCGCTTGTCCTCTTTGTT |
| *GBX2* | Forward | GACGAGTCAAAGGTGGAAGAC |
|  | Reverse | GATTGTCATCCGAGCTGTAGTC |
| *TH* | Forward | GGAAGGCCGTGCTAAACCT |
|  | Reverse | GTGGATTTTGGCTTCAAACG |
| *T/Brachyury* | Forward | CTATTCTGACAACTCACCTGCAT |
|  | Reverse | ACAGGCTGGGGTACTGACT |

**Table S3 PluriTest results**. The samples were analyzed using an algorithm that integrates bioinformatics to authenticate pluripotency potential. Samples are screened against samples in the stem cell matrix and given a pluripotency score (PluriCor) and novelty score (NovelCor), which are shown in the table. Pass shows clear pluripotency signature. Fail means the samples are not pluripotent. A non-iPSC sample was included in this experiment to serve as a negative control for non-pluripotency.

| **Sample** | **Sample ID** | **PluriTest Result** | **PluriCor** | **NovelCor** |
| --- | --- | --- | --- | --- |
| PT-415 | TCLab Orbital Shaker P3 | Pass | 40.81336 | 1.43566 |
| PT-416 | Gibco Orbital Shaker P3 | Pass | 40.08054 | 1.436489 |
| PT-418 | TCLab PBS P3 | Pass | 40.43361 | 1.522158 |
| PT-419 | GibcoEpi PBS P3 | Pass | 41.87922 | 1.41157 |
| PT-420 | iPSC control | Pass | 45.75131 | 1.326924 |
| PT-421 | Non-iPSC Control | Fail | -43.7921 | 2.756846 |


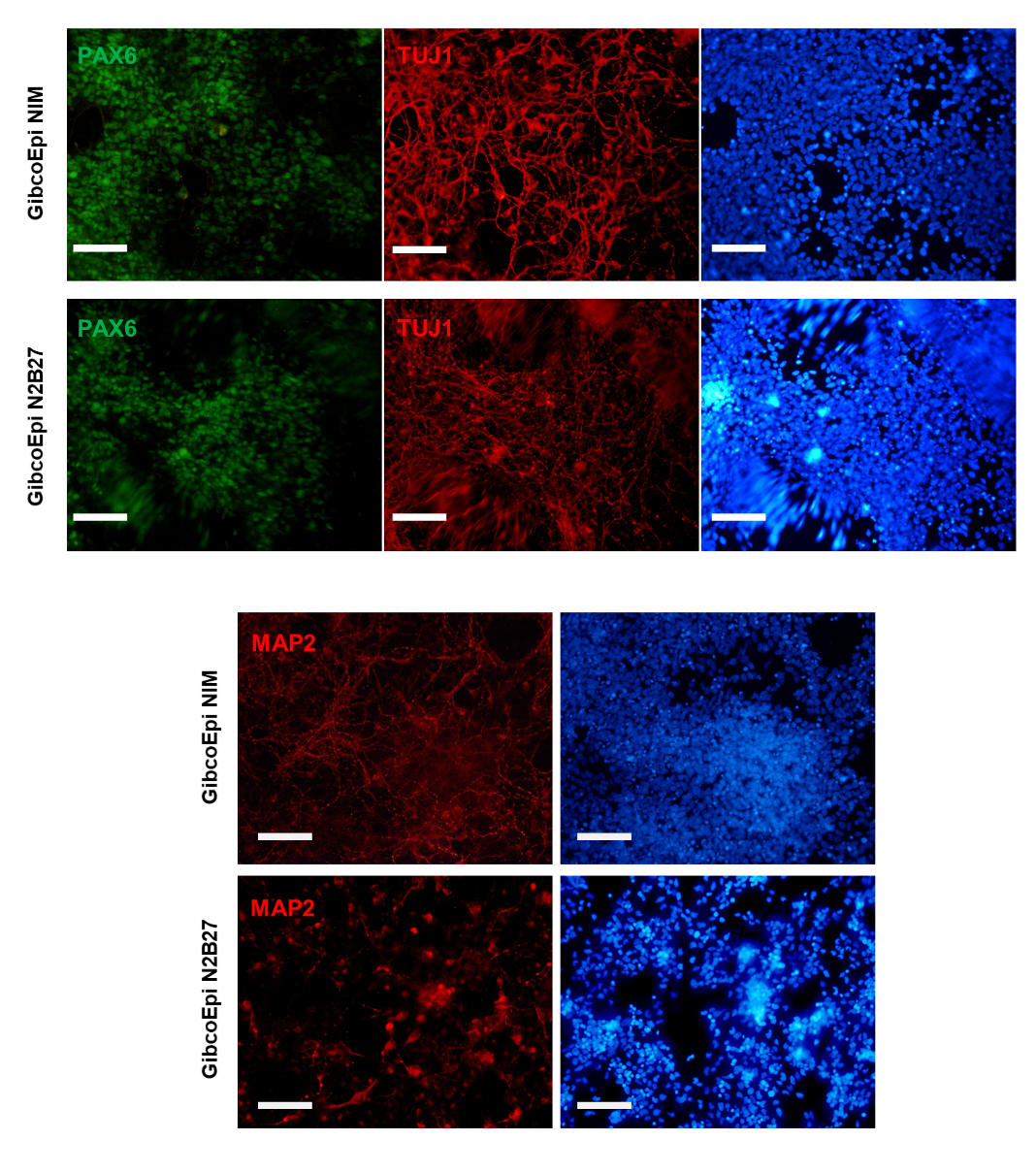


**Figure S1** Immunofluorescence for neural progenitor marker PAX6, neuron-specific microtubule-associated protein MAP2 and β-III-tubulin (TUJ1) of cells differentiated using Neural Induction Medium (NIM) and N2B27 at day 34 of differentiation. Scale bars: 100 µm.


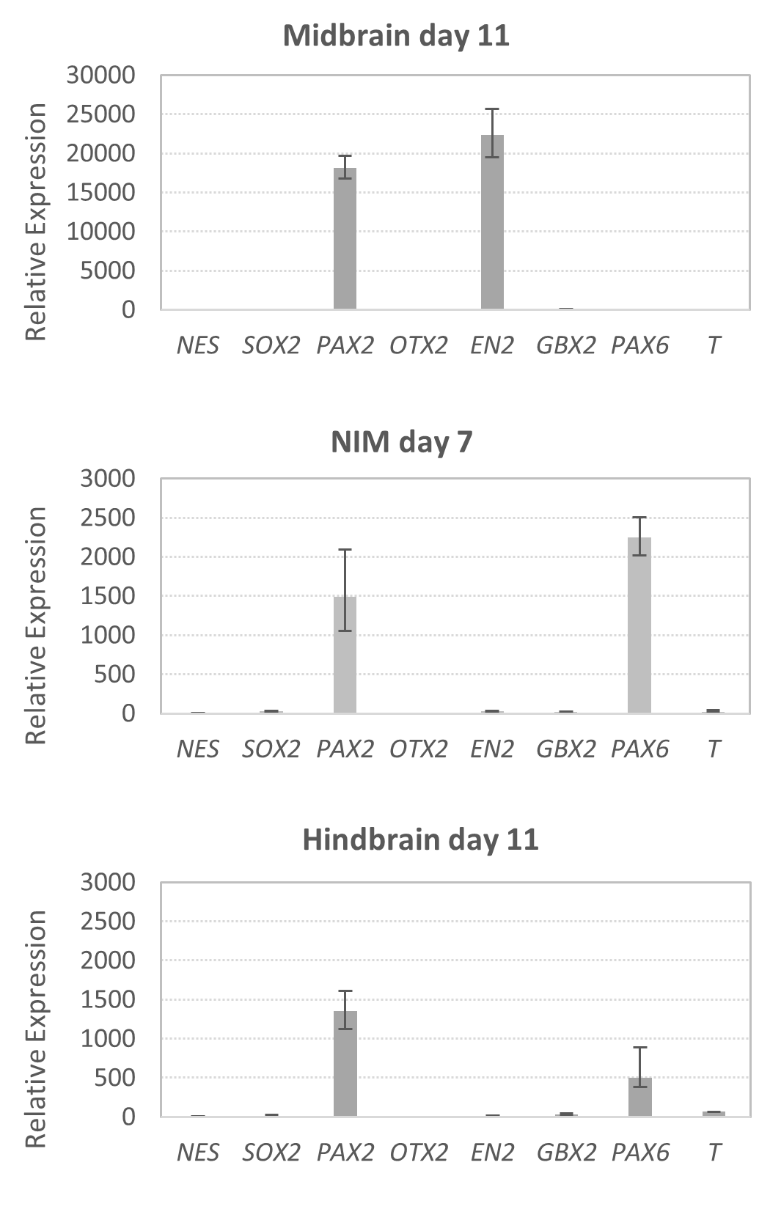


**Figure S2** qRT-PCR analysis of cells after hiPSC differentiation into different neural lineages. The cells were tested for Nestin (NES), SOX2, PAX2, OTX2, EN2, GBX2, PAX6, and T/BRACHYURY marker expression. Fold change is relative to expression of GAPDH.
